# Prostate cancer risk related to foods, food groups, macronutrients and micronutrients derived from the UK Dietary Cohort Consortium food diaries

**DOI:** 10.1038/ejcn.2016.162

**Published:** 2016-09-28

**Authors:** J A Lane, S E Oliver, P N Appleby, M A H Lentjes, P Emmett, D Kuh, A Stephen, E J Brunner, M J Shipley, F C Hamdy, D E Neal, J L Donovan, K-T Khaw, T J Key

**Affiliations:** 1 School of Social and Community Medicine, University of Bristol Bristol, UK; 2 NIHR Biomedical Research Unit in Nutrition, Diet and Lifestyle, Level 3, University Hospitals Bristol Education Centre, Bristol, UK; 3 University of York and Hull York Medical School, York, UK; 4 Cancer Epidemiology Unit, Nuffield Department of Population Health, University of Oxford, Oxford, UK; 5 Medical Research Council Centre for Nutritional Epidemiology in Cancer Prevention and Survival, Cambridge, UK; 6 Medical Research Council Unit for Lifelong Health and Ageing at UCL, London, UK; 7 Department of Nutritional Sciences, University of Surrey, Guildford, Surrey, UK; 8 Department of Epidemiology and Public Health, University College London, London, UK; 9 Nuffield Department of Surgical Sciences, University of Oxford, Oxford, UK; 10 Cambridge University and Cambridge University Hospitals NHS Trust, Cambridge, UK; 11 Department of Public Health and Primary Care, University of Cambridge, Cambridge, UK

## Abstract

**Background/Objectives:**

The influence of dietary factors remains controversial for screen-detected prostate cancer and inconclusive for clinically detected disease. We aimed to examine these associations using prospectively collected food diaries.

**Subjects/Methods:**

A total of 1,717 prostate cancer cases in middle-aged and older UK men were pooled from four prospective cohorts with clinically detected disease (*n*=663), with routine data follow-up (means 6.6–13.3 years) and a case-control study with screen-detected disease (*n*=1054), nested in a randomised trial of prostate cancer treatments (ISCTRN 20141297). Multiple-day food diaries (records) completed by men prior to diagnosis were used to estimate intakes of 37 selected nutrients, food groups and items, including carbohydrate, fat, protein, dairy products, fish, meat, fruit and vegetables, energy, fibre, alcohol, lycopene and selenium. Cases were matched on age and diary date to at least one control within study (*n*=3528). Prostate cancer risk was calculated, using conditional logistic regression (adjusted for baseline covariates) and expressed as odds ratios in each quintile of intake (±95% confidence intervals). Prostate cancer risk was also investigated by localised or advanced stage and by cancer detection method.

**Results:**

There were no strong associations between prostate cancer risk and 37 dietary factors.

**Conclusions:**

Prostate cancer risk, including by disease stage, was not strongly associated with dietary factors measured by food diaries in middle-aged and older UK men.

## Introduction

Prostate cancer is the most commonly detected life-threatening cancer among men in most Western countries, and accounted for over 300 000 deaths worldwide in 2012.^1^ The incidence of prostate cancer is increasing worldwide, largely due to screening programmes, and has doubled in the UK from 1984 to 2007.^2^ The established risk factors for prostate cancer are age, ethnicity, family history of the disease and some genetic factors.^3^ Increasingly, obesity has been linked to aggressive prostate cancer risk.^4^ Prostate cancer incidence and mortality varies globally, suggesting that diet and environmental factors may explain some of the geographic variation.^5^ Several hypotheses have been explored, including that prostate cancer risk may be elevated by diets rich in meat, dairy products or fat, and may be lowered by diets high in fibre, fruit, vegetables and various micronutrients.^5, 6^ The epidemiological evidence for selenium and vitamin E was judged sufficient to commence a randomised supplementation trial, but this was stopped early due to no benefit,^7^ with subsequent follow-up indicating an increased prostate cancer risk with vitamin E supplementation.^8^ The American Institute for Cancer Research/World Cancer Research Fund (AICR/WCRF) guidelines currently identify the carotenoid lycopene, a pigment found in tomatoes and other fruits as having a ‘probable' protective effect on prostate cancer risk,^5^ whereas diets rich in calcium were classed as ‘probably' increasing prostate cancer risk.

Epidemiological studies of diet and cancer have predominantly utilised food frequency questionnaires (FFQ) to measure intake.^9^ The greater measurement error of some dietary items associated with FFQs in comparison to measurement by multiple-day food diaries (records) has been suggested to account for some null findings for diet and cancer risk,^10, 11^ although this is contested.^12^

The UK Dietary Cohort Consortium was established in 2006^13^ to understand diet and cancer relationships, using up to eight population-based prospective studies with food diaries (records). We have utilised the consortium data to analyse prostate cancer risk in relation to dietary intake of food groups (meat, fish, dairy products, fruit and vegetables), macronutrients and micronutrients potentially associated with disease.

## Participants and methods

### Study population

Table 1 summarises the five UK Dietary Cohort Consortium studies that contributed data: European Prospective Investigation into Cancer and Nutrition (EPIC)-Norfolk,^14^ EPIC-Oxford,^15^ Medical Research Council National Survey of Health and Development (NSHD),^16^ Prostate testing for cancer and Treatment study (ProtecT^17^) and Whitehall II.^18^ Two additional cohorts only recruited females and one focused on vegetarians, so they were excluded from this analysis. The study designs, ethical approvals and conduct have been described in detail elsewhere.^14, 15, 16, 17, 18^ Information on demographic and lifestyle factors was collected either during participant interviews or by using questionnaires administered prior to, or contemporaneously with, the completion of the food diary.

### Ascertainment of prostate cancer and follow-up

Four prospective cohort studies (EPIC-Norfolk, EPIC-Oxford, NSHD and Whitehall II) obtained prostate cancer diagnoses through record linkage with the UK National Health Service Office for National Statistics and cancer registries. Case participants were individuals who were undiagnosed with cancer (except non-melanoma skin cancer) at the time of diary completion and who were diagnosed with prostate cancer at least 12 months later (6 months in EPIC-Oxford) but before the closure date for each cohort (latest date of complete follow-up for cancer incidence and vital status, which was the same for cases and controls) (Table 1). The ninth and tenth revisions of the International Statistical Classification of Diseases, Injuries and Causes of Death (ICD) were used to define prostate cancer (codes 185 or C61). Clinical staging data from cancer registries (where available) utilised the tumour, nodes and metastasis system, with T1-T2 (N0 or Nx, M0 or Mx) categorised as localised disease and T3-T4 as advanced disease; Gleason grade was unavailable in most cohorts, where cases were identified through routine data record linkage, so this clinical factor was excluded from all analyses.

ProtecT is an ongoing randomised controlled trial of treatments in men diagnosed with localised prostate cancer following the community-based prostate-specific antigen (PSA) testing in nine centres across the UK, which will publish trial outcomes in 2016 (ISCTRN20141297).^17^ Men aged 50–69 years registered at randomly selected general practices were invited to attend recruitment/PSA-testing clinics. There was no selection by symptoms or PSA status (13% had received a prior test) and the UK does not have a prostate cancer screening programme.^19^ Around 40% of invited men attended clinics between 2003 and 2009. Food diaries were distributed by trial nurses at recruitment to men also participating in the ProMPT (Prostate Mechanisms of Progression and Treatment) translational study, with over 75% returned prior to receipt of PSA results. Participants with an elevated PSA result (⩾3.0 ng/ml, a threshold linked to contemporary clinical practice in the UK) underwent 10-core prostate biopsies (87% of those eligible received a biopsy) and those with a negative biopsy were offered a second biopsy.

### Selection of matched controls

Cases were matched within an individual study with up to four control participants selected at random from all eligible potential controls within the matching criteria. Cohort controls were men without notified prostate cancer at closure date for follow-up, whereas ProtecT controls either had a PSA result of <3.0 ng/ml or negative prostatic biopsies. Matching criteria within each study were age (generally ±3 years from diary commencement, ±6 months for EPIC-Oxford and ±5 years for ProtecT) and diary completion date (generally ±3 months, ±6 months for EPIC-Oxford).

### Measurement of food and nutrient intake

Seven-day food diaries (five-day in NSHD) were completed at recruitment (EPIC-Norfolk and ProtecT) or ~6 months later (EPIC-Oxford) or at second follow-up (Whitehall II) or when participants were 43 years old (NSHD). Participants were asked to record all food and drinks consumed at the times specified (for example, breakfast and lunch), with photographs of food items to aid estimation of portion sizes. Information from food diaries was coded to derive nutrient intakes based on national food composition tables contemporaneous with diary completion dates as described previously.^13^ The food groups were defined by the consortium: red meat, processed meat, poultry, white and oily fish, and included disaggregation of dishes containing constituent foods;^20^ additional food groups studied were yogurt, cheese and milk, which were also used to calculate dairy protein intake (sum of those products, dairy creams, chocolate and milk drinks, ice cream, dairy sauces, chocolate desserts and other animal milks). EPIC-Norfolk, EPIC-Oxford, Whitehall II and 1107 ProtecT diaries were coded with the Data Into Nutrients for Epidemiological Research (DINER) data entry^21^ and Data Into Nutrients for Epidemiological Research Moving Onwards (DINERMO) processing software,^22^ whereas NSHD and 1208 of the ProtecT diaries (coded before joining the consortium but case/control pairs coded with the same software) used the Diet In Data Out (DIDO) programme.^23^ Some NSHD (100) and ProtecT (99) diaries were processed with DIDO and DINERMO, and there was good agreement for total energy, carbohydrate, fat, calcium, total sugars and starch intakes. The DIDO programme gave lower values for alcohol intake than DINERMO, which we hypothesised reflected UK alcohol measures having increased over time, so the DIDO estimates were retained as they were determined contemporaneously to diary completion.

### Statistical methods

The pre-specified consortium analysis plan for all cancers defined the selection and categorisation of dietary exposures and confounders with lycopene and selenium added to the prostate plan based on AICR/WCRF guidelines for prostate cancer prevention.^5^ Analyses used all available data; all tests were two sided with no adjustment for multiple comparison and included diaries with incomplete days. No exclusions were made on the basis of reported levels of energy intake; only 16 (0.3%) participants fell outside recommended cutoffs (<800 kcal or >4000 kcal in men).^24^ Conditional logistic regression was used to calculate odds ratios (ORs) and 95% confidence intervals (95% CI) for prostate cancer risk according to quintiles of intake of 37 dietary variables (quintiles calculated on intakes combined across studies for all participants), with the *P* value for trend test being of principal interest. There was a high proportion of non-consumers of oily fish (first and second quintiles were combined) and yoghurt (first three combined), whereas five pre-specified cut-points were used for alcohol consumption (<1, 1–9, 10–19, 20–39, 40 and above g/d). To test for trends in prostate cancer risk across the distribution of intakes, we calculated the ORs (95% CI) for a 1 s.d. increase in nutrient intake with the *P* value being obtained by comparing the ratio of the logarithm of the OR and its standard error to the normal distribution.

As age is a risk factor for prostate cancer, age was utilised as a continuous variable in the regression models. Additional adjustment was made for other potential confounders, that is, total energy intake (quintiles), body mass index (BMI: <22.5, 22.5–24.9, 25.0–27.4, 27.5–29.9, 30.0 and above, unknown kg/m^2^), smoking status (never, previous, current, unknown), marital status (married or cohabiting, single including divorced and separated, unknown), self-reported diabetes at recruitment (no, yes, unknown) and a residential area-based measure of material deprivation (quartiles of Townsend Score).^25^ Unknown values were categorised as missing (BMI 6%, smoking 5%, marital status 1%, diabetes 8% and socioeconomic measure 3%). The majority of epidemiological evidence on diet and prostate cancer risk relates to studies in which cases were identified clinically, and to enable comparison with this pre-existing literature disease risk was also assessed for the cohort studies combined (that is, predominantly clinically diagnosed disease) and for PSA-detected disease (ProtecT study, akin to screening) and reported as risk per 1 s.d. increase in dietary intake. Disease-diet associations were also examined and reported in the same way for localised and advanced prostate cancer. Analyses were performed using Stata version 10.^26^

## Results

### Study and participant characteristics

In total, 1717 men diagnosed with prostate cancer were compared with 3528 matched controls without prostate cancer (Table 1). There were 1277 cases of localised prostate cancer (74.4%) and 226 advanced cases (13.2%), whereas for 214 cases the disease stage at diagnosis was unknown (12.5%). Table 2 summarises the clinical and socio-demographics of participants by prostate cancer status. Participants had a mean age of 62 years at recruitment, were slightly overweight on average (BMI 26.3 kg/m^2^) and over 85% were married or cohabiting.

### Dietary intake and overall prostate cancer risk

The unadjusted intakes of dietary factors for cases and controls combined for the five studies are shown in Table 3. There were some modest differences in consumption between cases and controls; namely, oily fish, red meat and protein (each 2% more in cases), energy (1.5% less), cheese (3% less), yoghurt (12% more), alcohol (4% more), fruit and vegetables (1% less), vitamin C (2% more), calcium (1% more), retinol (1% less) and selenium (4% more). The adjusted risk estimates for overall prostate cancer incidence showed no statistically significant linear trends across the distributions of the 37 dietary factors (Table 4).

### Dietary intake and risk of prostate cancer by detection method and disease stage

The risk of prostate cancer detected clinically or by PSA in relation to dietary intakes is shown in Table 5. There was no significant heterogeneity in associations according to method of diagnosis, except for vitamin D, but vitamin D was not significantly associated with risk for either clinically or PSA-detected cancer.

The risk of prostate cancer across food and nutrient groups (Table 6) shows that there was no significant heterogeneity for any of the foods or nutrients by disease stage.

## Discussion

Prostate cancer risk in middle-aged and older men was not associated with any of the 37 dietary components examined in this comprehensive analysis based on food diaries (records). There was weak evidence of heterogeneity of risk for vitamin D between clinically and screen-detected disease, but this finding may be due to chance. The main strengths of this study are its size and diversity through pooling over 1700 prostate cancer cases from five predominantly population-based UK studies with adjustment for clinical and demographic confounders and the capacity to compare clinically and screen-detected estimates of risk. Dietary records were completed prior to men's knowledge of disease status in the prospective cohorts, or prior to biopsies in ProtecT, thus excluding recall bias.

This evaluation of prostate cancer risk and dietary factors is also one of the few studies to examine intakes derived from food diaries rather than FFQs. Biomarker validation studies have shown that food diaries are more accurate than FFQs for estimating some nutrients.^27, 28^ Pooling five studies may have potentially introduced non-differential errors in nutrient intakes across the studies, but the consortium provided training, protocols and data-checking software to enhance consistency. We collected data on entire cohorts and utilised a nested matched case-control analysis to accommodate the resources required for diary coding, but this reduced the power to identify weak associations compared with a complete cohort analysis.

Limitations of these analyses include the inability to adjust for individual social class, which potentially created a confounder in the cohort studies as prostate cancer testing is more frequent in affluent individuals.^29^ Prostate cancer screening history was unavailable for the cohorts, although PSA testing rates are probably low as there is no formal UK screening programme (UK figures are 4–6%^19, 30^) and less than 15% had received a prior test in the ProtecT study.^17^ All ProtecT controls with a PSA ⩾3.0 ng/ml had a negative biopsy result, thus reducing misattribution bias (disease risk increases with PSA values), but the absence of pathological confirmation of disease-free status for the majority of these controls is an unavoidable limitation, which might attenuate diet and prostate cancer associations because some controls will have undiagnosed disease (based on autopsy data^31^). Clinical stage was missing for the NHSD and Whitehall studies, which reduced the power to examine differences by stage (although they contributed fewest cases), and it was impossible to examine the associations of diet subdivided by Gleason grade. Some differences (for example, diary duration) could not be rectified in the analysis, as these studies were established before the diet consortium, and some confounders relevant to prostate cancer were not collected in all studies, for example, family history of cancer, or were measured in ways that did not allow pooling (for example, physical activity). We utilised standardised dietary coding systems, which increased exposure quantification consistency, although heterogeneity in measurement duration could have also potentially modified any associations. All dietary data instruments have limitations, which we aimed to minimise where possible; differential misclassification through using a prospective design and food diaries to reduce measurement error, although some non-differential misclassification will exist for estimated diet constituents. Participants were predominantly white, thus potentially limiting the wider generalisability to other ethnic populations.

A recent meta-analysis of dietary factors and supplements and prostate cancer risk has concluded that the intake of red and well-done meat, fat and milk should be limited, whereas lycopene, green tea and potentially soy-containing products may be preventative.^6^ These dietary components were not associated in this study with clinically or screen-detected disease, or with disease stage (green tea and soy products were not evaluated). However, recent evidence that ProtecT participants who consumed at least 10 portions of tomatoes weekly showed an 18% reduced risk of developing prostate cancer supports the meta-analyses recommendations.^32^ Previously, the EPIC consortium found an increased prostate cancer risk with the highest quartiles of dairy protein,^33^ but no association with dietary fat (mostly using FFQs).^32, 34^ Data from the US Health Professionals study based on clinically detected cases found no association between calcium intake and localised prostate cancer (measured with FFQs) but a positive association with advanced disease.^35^ Conversely, calcium intake was related to an increased risk of localised disease with screen-detected cases in the US PLCO trial.^36^

The evidence for a link between obesity and fatal prostate cancer^4^ is strengthening and energy intake might be on that causal pathway. An association between energy intake and advanced disease was shown in a meta-analysis for studies with disease stage with a combined odds ratio of 1.6 for advanced disease.^37^ In this study, there was no overall relationship between energy intake and prostate cancer nor heterogeneity in the risk of disease by stage (*P*=0.07); the association with advanced disease was positive (23% increase) but did not reach conventional statistical significance (95% CI 1.00–1.51).

The finding of weak evidence of heterogeneity in the association of vitamin D with risk between clinically and screen-detected disease may merit further investigation. The precision of estimates of foods consumed irregularly, such as oily fish, a good source of vitamin D, may be lower in food diaries than in questionnaires. Vitamin D levels are also related to sunlight exposure, making serological assessments more comprehensive. In the ProtecT study, deficiency in vitamin D (circulating concentration <12 ng/ml) was associated with a greater risk of aggressive prostate cancer (higher grade or stage),^38^ which would be more prevalent in clinically detected cases, but the recent meta-analysis does not support vitamin D supplementation, except for deficiency.^6^

There was no association of overall diet (assessed using FFQs) and screen-detected prostate cancer in the US PCPT trial nor in the Swedish study.^39, 40^ Food diary data from 133 prostate cancer cases also revealed no association with diet and prostate cancer, but a reduction with a Mediterranean-style diet rich in monounsaturated fatty acids and vegetables/fruits and low in red meats.^41^ A recent meta-analysis of adherence to a Mediterranean diet and overall cancer risk showed a 4% risk reduction for prostate cancer incidence.^42^

The natural history of prostate cancer remains poorly understood, including the time points when dietary and environmental factors may influence disease development or progression.^43^ This study measured dietary intake prior to diagnosis and found no major associations with prostate cancer risk, yet migrant studies and international variation in prostate cancer incidence suggest that dietary or other environmental components contribute to disease risk. More recent evidence highlights a role of dietary factors in disease progression, for example, fat intake may influence prostate cancer mortality.^44^ Future studies will need to extend measurement of dietary intake across the life course, consider intermediary influences such as the insulin-like growth factor axis and examine the role of obesity, which increases the risk of aggressive prostate cancer, subsequent disease progression and mortality.^4^

## Conclusions

In summary, this large study revealed no strong evidence that prostate cancer risk is associated with dietary intake measured prior to diagnosis in middle-aged and older men.

## Figures and Tables

**Table 1:** Characteristics of the Dietary Cohort Consortium studies

*Study*^a^	*Participants*	*Diary completion (years)*	*Final follow-up date*	*Follow-up duration (years)*^b^	*Prostate cancer cases (*n)	*Clinical stage (*n*, advanced/localised/ unknown)*	*Controls (*n)	*Control matching*		*Age at diary completion (years)*^b^
								*Age at diary completion*	*Month of diary completion*	
EPIC-Norfolk	Population	1993–1998	31/12/2009	7.3 (3.2)	439	105/251/83	1752	± 3 years	± 3 months	64.8 (7.7)
EPIC-Oxford	Population and vegetarians	1993–1999	31/12/2007	6.6 (2.7)	125	22/73/30	125	± 6 months	± 6 months	64.6 (8.0)
NSHD	Born 1946	1989–1990	31/12/2008	13.3 (3.3)	15	0/0/15	60	± 3 years	± 3 months	43.5 (0.2)
ProtecT	Population	2003–2009	29/04/2009	0.2 (0.3)	1054	99/953/2	1261	± 5 years	± 3 months	62.9 (4.7)
Whitehall II	Civil servants	1991–1993	29/11/2005	9.0 (2.9)	84	0/0/84	330	± 3 years	± 3 months	54.8 (4.8)

a Abbreviations: EPIC, European Prospective Investigation into Cancer and Nutrition; NSHD, National Survey of Health and Development; ProtecT, Prostate testing for cancer and Treatment.

b Mean (s.d.) in years.

**Table 2:** Baseline characteristics of prostate cancer cases and controls pooled across five studies

*Characteristic*^a^	*Controls (*n=*3528)*	*Cases (*n=*1717)*
Age at diary completion (years)	62.7 (7.5)	63.0 (6.5)
Height (m)^b^	1.75 (0.07)	1.75 (0.07)
Weight (kg)^c^	80.7 (11.6)	80.8 (11.7)
Body mass index (kg/m^2^)	26.4 (3.3)	26.3 (3.3)
		
*Body mass index, categories,* n*(% known)*		
<22.5	334 (9.9)	171 (10.8)
22.5–24.9	822 (24.5)	428 (27.1)
25.0–27.4	1100 (32.7)	487 (30.8)
27.5–29.9	643 (19.1)	291 (18.4)
⩾30.0	462 (13.7)	203 (12.8)
Missing, *n* (% all)	167 (4.7)	137 (8.0)
		
*Smoking status,* n*(% known)*		
Never	1116 (33.1)	605 (37.7)
Former	1873 (55.5)	821 (51.2)
Current	383 (11.4)	177 (11.0)
Missing, *n* (% all)	156 (4.4)	114 (6.6)
		
*Marital status, n (% known)*		
Married or cohabiting	3030 (86.4)	1500 (88.0)
Unmarried	478 (13.6)	205 (12.0)
Missing, *n* (% all)	20 (0.6)	12 (0.7)
		
*Diabetes, n (% known)*		
No diabetes	3121 (94.4)	1462 (95.1)
Diabetes (self-reported)	185 (5.6)	76 (4.9)
Missing, *n* (% all)	222 (6.3)	179 (10.4)
		
*Townsend material deprivation score, n (% known)*		
Low (richest)	817 (24.6)	403 (25.5)
Medium-low	864 (26.0)	370 (23.4)
Medium-high	837 (25.2)	383 (24.3)
High (poorest)	802 (24.2)	422 (26.7)
Missing, *n* (% all)	208 (5.9)	139 (8.1)

a Values are unadjusted means (s.d. except where indicated) combined for five studies.

b Missing for 151 (4.3%) controls, 115 (6.7%) cases.

c Missing for 157 (4.5%) controls, 131 (7.6%) cases.

**Table 3:** Consumption of food groups, foods, macronutrients and micronutrients pooled across five studies

*Dietary intake and units*^a^	*Controls (*n=*3528)*	*Prostate cancer cases (*n=*1717)*
*Food groups*		
Red meat (g/d)	41.6 (31.3)	42.3 (31.2)
Processed meat (g/d)	27.8 (22.0)	27.6 (21.2)
Red and processed meat (g/d)	69.4 (39.8)	70.0 (39.8)
Poultry (g/d)	25.7 (24.8)	26.0 (25.6)
White fish (g/d)	15.9 (17.2)	15.3 (17.7)
Oily fish (g/d)	14.7 (21.2)	15.6 (22.8)
Milk (g/d)	207 (143)	205 (146)
Cheese (g/d)	17.4 (17.2)	16.0 (17.2)
Yogurt (g/d)	24.1 (43.2)	26.9 (47.0)
Fruit and vegetables (172 g/d)	313 (174)	310 (169)
Total energy intake (MJ/d)	9.12 (2.11)	8.98 (2.07)
		
*Macronutrients*		
Protein (% energy)	15.4 (2.6)	15.7 (2.7)
Protein from dairy products (% energy)	2.6 (1.3)	2.6 (1.4)
Carbohydrate (% energy)	45.6 (6.7)	45.2 (6.9)
Total fat (% energy)	33.2 (5.4)	32.9 (5.5)
Saturated fat (% energy)	12.4 (3.0)	12.1 (3.1)
Monounsaturated fat (% energy)	11.5 (2.1)	11.4 (2.1)
Polyunsaturated fat (% energy)	6.2 (1.7)	6.1 (1.8)
n-6 fatty acids (% energy)*	5.3 (1.8)	5.2 (1.7)
n-3 fatty acids (% energy)*	0.69 (0.26)	0.71 (0.30)
Ratio n-6:n-3^b^	8.4 (3.7)	8.2 (3.8)
Alcohol (g/d)	18.4 (21.4)	19.2 (21.9)
Dietary fibre (g/d)	15.9 (6.0)	15.6 (6.0)
		
*Micronutrients*		
Retinol (μg/d)	700 (1072)	656 (1020)
Carotene (μg/d)*	2675 (1556)	2773 (1597)
Lycopene (μg/d)*	1485 (1983)	1481 (1968)
Vitamin B6 (mg/d)	2.25 (0.66)	2.31 (0.68)
Folate (μg/d)	293 (90)	295 (89)
Vitamin B12 (μg/d)	5.68 (3.97)	5.64 (3.96)
Vitamin C (mg/d)	87.7 (51.6)	89.2 (55.0)
Vitamin D (μg/d)	3.82 (2.76)	3.88 (2.80)
Vitamin E (mg/d)	11.0 (4.9)	10.8 (5.0)
Calcium (mg/d)	896 (283)	887 (283)
Iron (mg/d)	13.1 (4.0)	13.0 (3.9)
Magnesium (mg/d)	322 (91)	323 (90)
Selenium (μg/d)	71.0 (31.4)	73.8 (40.1)
Zinc (mg/d)	9.52 (2.53)	9.52 (2.55)

a Values are unadjusted means or percentages (s.d.).

b Unknown for some participants.

**Table 4:** Odds ratios for prostate cancer diagnosis by food groups, foods and nutrient consumption

*Food group, food or nutrient*^a^	*Food or nutrient intake (increasing quintiles except where indicated)*					P *value for trend*^b^
	*1 (referent)*	*2*	*3*	*4*	*5*	
*Red meat (g/d)*						
Cut-point		14.6	30.4	45.4	65.3	
Cases/controls	345/688	330/735	331/718	355/694	356/693	
Odds ratio (95% CI)	1.00	0.90 (0.74–1.09)	0.93 (0.77–1.14)	1.02 (0.84–1.24)	0.99 (0.81–1.21)	0.99
						
*Processed meat (g/d)*						
Cut-point		8.6	18.9	29.3	43.7	
Cases/controls	333/716	342/710	347/699	340/709	355/694	
Odds ratio (95% CI)	1.00	1.06 (0.87–1.29)	1.10 (0.91–1.34)	1.11 (0.91–1.35)	1.14 (0.93–1.39)	0.98
						
*Red and processed meat (g/d)*						
Cut-point		37.2	56.8	75.9	99.7	
Cases/controls	344/705	341/708	321/728	349/700	362/687	
Odds ratio (95% CI)	1.00	1.03 (0.84–1.26)	0.95 (0.78–1.16)	1.07 (0.88–1.31)	1.05 (0.86–1.29)	0.99
						
*Poultry (g/d)*						
Cut-point		0.2	15.3	27.1	43.2	
Cases/controls	388/718	312/687	323/716	339/713	355/694	
Odds ratio (95% CI)	1.00	0.86 (0.71–1.05)	0.87 (0.72–1.06)	0.89 (0.74–1.08)	0.95 (0.79–1.15)	0.78
						
*White fish (g/d)*						
Cut-point		0.2	9.3	16.5	27.1	
Cases/controls	626/1224	86/161	351/708	286/690	368/745	
Odds ratio (95% CI)	1.00	1.04 (0.77–1.40)	1.02 (0.86–1.21)	0.92 (0.77–1.10)	1.10 (0.93–1.31)	0.54
						
*Oily fish (g/d)*^c^						
Cut-point		—	0.2	12.9	28.6	
Cases/controls	788/1603	—	213/511	350/728	366/686	
Odds ratio (95% CI)	1.00	—	0.89 (0.73–1.08)	0.93 (0.79–1.10)	1.00 (0.85–1.18)	0.83
						
*Milk (g/d)*						
Cut-point		89	154	216	308	
Cases/controls	349/699	343/707	350/699	340/709	335/714	
Odds ratio (95% CI)	1.00	1.05 (0.87–1.28)	1.05 (0.86–1.27)	1.04 (0.86–1.27)	1.04 (0.85–1.28)	0.33
						
*Cheese (g/d)*						
Cut-point		2.6	9.9	16.5	28.4	
Cases/controls	372/674	364/687	340/710	326/733	315/724	
Odds ratio (95% CI)	1.00	1.04 (0.86–1.26)	0.95 (0.78–1.15)	0.89 (0.73–1.08)	0.95 (0.77–1.16)	0.25
						
*Yogurt (g/d)*^d^						
Cut-point		—	—	0.4	49.3	
Cases/controls	1005/2149	—	—	350/690	362/689	
Odds ratio (95% CI)	1.00	—	—	1.09 (0.93–1.28)	0.92 (0.79–1.08)	0.57
						
*Fruit and vegetables (g/d)*						
Cut-point		171	246	325	434	
Cases/controls	343/706	357/692	334/715	336/713	347/702	
Odds ratio (95% CI)	1.00	1.10 (0.91–1.34)	0.99 (0.81–1.21)	1.04 (0.85–1.27)	1.05 (0.85–1.28)	0.66
						
*Energy (MJ/d)*						
Cut-point		7.33	8.45	9.47	10.76	
Cases/controls	362/687	366/683	340/709	320/729	329/720	
Odds ratio (95% CI)	1.00	1.09 (0.90–1.32)	1.00 (0.82–1.21)	0.97 (0.79–1.18)	1.11 (0.91–1.36)	0.72
						
*Protein (% energy)*						
Cut-point		13.3	14.7	15.9	17.5	
Cases/controls	306/743	318/731	363/686	352/697	378/671	
Odds ratio (95% CI)	1.00	1.00 (0.82–1.23)	1.16 (0.95–1.42)	1.02 (0.83–1.25)	1.03 (0.83–1.29)	0.68
						
*Dairy protein (% energy)*						
Cut-point		1.5	2.1	2.7	3.5	
Cases/controls	372/677	326/723	332/717	331/718	356/693	
Odds ratio (95% CI)	1.00	0.88 (0.73–1.07)	0.89 (0.73–1.08)	0.91 (0.75–1.11)	0.97 (0.80–1.18)	0.95
						
*Carbohydrate (% energy)*						
Cut-point		39.9	44.2	47.3	51.0	
Cases/controls	358/691	357/692	346/703	323/726	333/716	
Odds ratio (95% CI)	1.00	1.03 (0.85–1.25)	1.03 (0.85–1.25)	0.93 (0.76–1.13)	1.04 (0.85–1.28)	0.59
						
*Total fat (% energy)*						
Cut-point		28.6	31.8	34.6	37.5	
Cases/controls	368/681	342/707	340/709	338/711	329/720	
Odds ratio (95% CI)	1.00	0.95 (0.78–1.15)	0.98 (0.81–1.19)	0.99 (0.81–1.21)	1.00 (0.82–1.21)	0.83
						
*SFA (% energy)*						
Cut-point		9.9	11.3	12.8	14.6	
Cases/controls	384/665	336/713	343/706	323/726	331/718	
Odds ratio (95% CI)	1.00	0.96 (0.79–1.16)	1.03 (0.85–1.25)	1.01 (0.83–1.23)	1.04 (0.85–1.27)	0.98
						
*MUFA (% energy)*						
Cut-point		9.8	10.9	12.0	13.1	
Cases/controls	353/696	349/700	347/702	329/720	339/710	
Odds ratio (95% CI)	1.00	1.07 (0.89–1.30)	1.08 (0.89–1.31)	1.07 (0.88–1.30)	1.04 (0.86–1.26)	0.67
						
*PUFA (% energy)*						
Cut-point		4.8	5.6	6.4	7.4	
Cases/controls	367/682	333/716	343/706	347/702	327/722	
Odds ratio (95% CI)	1.00	0.93 (0.77–1.13)	1.04 (0.85–1.26)	1.02 (0.84–1.24)	0.98 (0.80–1.19)	0.78
						
*n-6 fatty acids (% energy)*^e^						
Cut-point		4.0	4.7	5.4	6.5	
Cases/controls	235/593	232/596	254/573	239/589	216/611	
Odds ratio (95% CI)	1.00	0.91 (0.72–1.14)	1.04 (0.82–1.31)	0.98 (0.78–1.24)	0.86 (0.68–1.09)	0.42
						
*n-3 fatty acids (% energy)*^e^						
Cut-point		0.51	0.60	0.71	0.86	
Cases/controls	246/582	213/615	234/593	231/597	252/575	
Odds ratio (95% CI)	1.00	0.80 (0.64–1.01)	0.91 (0.72–1.14)	0.85 (0.68–1.07)	0.91 (0.72–1.14)	0.72
						
*Ratio n-6:n-3*^e^						
Cut-point		5.5	6.8	8.3	10.7	
Cases/controls	243/585	263/565	228/599	223/605	219/608	
Odds ratio (95% CI)	1.00	1.15 (0.92–1.44)	0.99 (0.79–1.24)	0.93 (0.74–1.17)	0.94 (0.74–1.18)	0.75
						
*Alcohol (g/d)*^f^						
Cut-point		1.0	10.0	20.0	40.0	
Cases/controls	362/780	389/871	348/623	374/790	244/464	
Odds ratio (95% CI)	1.00	0.98 (0.81–1.18)	1.07 (0.88–1.30)	0.93 (0.77–1.12)	1.02 (0.82–1.28)	0.93
						
*Dietary fibre (g/d)*						
Cut-point		10.9	13.6	16.3	20.1	
Cases/controls	360/689	335/714	342/707	351/698	329/720	
Odds ratio (95% CI)	1.00	0.93 (0.76–1.13)	0.89 (0.73–1.10)	0.98 (0.80–1.21)	0.90 (0.72–1.12)	0.34
						
*Retinol (μg/d)*						
Cut-point		234	325	439	654	
Cases/controls	359/690	351/698	337/712	334/715	336/713	
Odds ratio (95% CI)	1.00	0.98 (0.81–1.19)	0.94 (0.77–1.15)	1.00 (0.81–1.24)	1.07 (0.86–1.33)	0.51
						
*Carotene (μg/d)*^e^						
Cut-point		1470	2139	2796	3696	
Cases/controls	234/593	230/599	223/604	237/591	252/575	
Odds ratio (95% CI)	1.00	0.96 (0.76–1.20)	0.88 (0.69–1.11)	0.95 (0.75–1.20)	0.96 (0.76–1.22)	0.84
						
*Lycopene (μg/d)*^e^						
Cut-point		350	775	1303	2140	
Cases/controls	217/596	236/577	258/554	237/576	213/599	
Odds ratio (95% CI)	1.00	1.10 (0.88–1.38)	1.17 (0.94–1.47)	1.02 (0.81–1.28)	0.85 (0.67–1.07)	0.28
						
*Vitamin B-6 (mg/d)*						
Cut-point		1.72	2.04	2.34	2.76	
Cases/controls	306/743	339/710	347/713	338/700	387/662	
Odds ratio (95% CI)	1.00	1.12 (0.91–1.37)	1.16 (0.94–1.42)	1.09 (0.88–1.35)	1.26 (1.00–1.58)	0.20
						
*Folate (μg/d)*						
Cut-point		218	261	304	362	
Cases/controls	336/713	338/711	327/722	369/680	347/702	
Odds ratio (95% CI)	1.00	1.00 (0.82–1.21)	1.03 (0.84–1.26)	1.21 (0.98–1.49)	1.04 (0.83–1.30)	0.69
						
*Vitamin B-12 (μg/d)*						
Cut-point		3.18	4.17	5.24	7.15	
Cases/controls	338/711	345/704	340/709	356/693	338/711	
Odds ratio (95% CI)	1.00	1.02 (0.84–1.24)	0.99 (0.81–1.21)	1.04 (0.85–1.27)	1.03 (0.83–1.26)	0.42
						
*Vitamin C (mg/d)*						
Cut-point		45.6	65.0	88.6	125.2	
Cases/controls	346/703	343/706	333/716	331/718	364/685	
Odds ratio (95% CI)	1.00	1.06 (0.87–1.28)	0.95 (0.78–1.16)	0.99 (0.81–1.21)	1.05 (0.86–1.29)	0.63
						
*Vitamin D (μg/d)*						
Cut-point		1.85	2.73	3.76	5.26	
Cases/controls	334/715	346/703	340/709	347/702	350/699	
Odds ratio (95% CI)	1.00	1.13 (0.93–1.37)	1.09 (0.90–1.34)	1.06 (0.87–1.30)	1.09 (0.88–1.33)	0.84
						
*Vitamin E (mg/d)*						
Cut-point		7.1	9.0	11.1	14.1	
Cases/controls	381/668	336/713	325/724	334/715	341/708	
Odds ratio (95% CI)	1.00	0.90 (0.74–1.09)	0.89 (0.73–1.09)	0.90 (0.73–1.11)	1.02 (0.81–1.27)	0.55
						
*Calcium (mg/d)*						
Cut-point		659	798	928	1112	
Cases/controls	362/687	337/712	328/721	366/683	324/725	
Odds ratio (95% CI)	1.00	0.98 (0.80–1.19)	0.96 (0.78–1.17)	1.20 (0.97–1.49)	1.00 (0.79–1.28)	0.53
						
*Iron (mg/d)*						
Cut-point		9.9	11.7	13.6	15.9	
Cases/controls	366/683	335/714	334/715	348/702	334/714	
Odds ratio (95% CI)	1.00	0.92 (0.75–1.12)	0.92 (0.75–1.14)	1.01 (0.81–1.26)	0.97 (0.76–1.24)	0.97
						
*Magnesium (mg/d)*						
Cut-point		248	292	334	390	
Cases/controls	339/710	358/691	316/733	352/697	352/697	
Odds ratio (95% CI)	1.00	1.10 (0.90–1.34)	0.90 (0.73–1.11)	0.99 (0.80–1.24)	1.02 (0.79–1.31)	0.63
						
*Selenium (μg/d)*						
Cut-point		49.3	61.0	73.2	89.1	
Cases/controls	319/730	316/733	376/673	335/714	371/678	
Odds ratio (95% CI)	1.00	0.93 (0.76–1.14)	1.19 (0.98–1.46)	0.93 (0.76–1.15)	0.95 (0.76–1.19)	0.95
						
*Zinc (mg/d)*						
Cut-point		7.4	8.7	9.8	11.4	
Cases/controls	347/702	327/722	369/681	341/707	333/716	
Odds ratio (95% CI)	1.00	0.94 (0.77–1.15)	1.07 (0.87–1.32)	0.93 (0.74–1.15)	0.89 (0.70–1.14)	0.77

a Conditional logistic regression adjusted for age, BMI, socioeconomic, smoking and marital status, diabetes and energy intake.

b *P* values relate to tests for trend obtained for continuous intake variable.

c First and second quintiles (and third^d^)

d combined due to large proportion of non-consumers.

e Unknown for some participants.

f Alcohol intake in five categories (<1, 1–9, 10–19, 20–39, ⩾40 g/d).

**Table 5:** Odds ratios for prostate cancer diagnosis with dietary intake by cancer detection method

*Food or nutrient intake (1 s.d.*)^a^	*All studies N=1717/3258*^b^	*Clinically-detected(4 studies) N=663/2267*^b^	*PSA-detected (ProtecT study) N=1054/1261*^b^	P *value for heterogeneity*^c^
Red meat (31.3 g/d)	1.00 (0.94–1.07)	0.96 (0.87–1.06)	1.04 (0.95–1.13)	0.25
Processed meat (21.8 g/d)	1.00 (0.94–1.07)	0.98 (0.89–1.08)	1.02 (0.94–1.11)	0.55
Red and processed meat (39.8 g/d)	1.00 (0.94–1.07)	0.96 (0.86–1.06)	1.04 (0.96–1.14)	0.20
Poultry (25.1 g/d)	1.01 (0.95–1.07)	1.05 (0.96–1.15)	0.98 (0.90–1.06)	0.27
White fish (17.4 g/d)	1.02 (0.96–1.08)	1.02 (0.93–1.11)	1.02 (0.93–1.11)	0.99
Oily fish (21.7 g/d)	1.01 (0.95–1.07)	0.94 (0.86–1.03)	1.06 (0.97–1.15)	0.08
Milk (144 g/d)	1.03 (0.97–1.10)	1.06 (0.97–1.16)	1.00 (0.91–1.10)	0.37
Cheese (17.2 g/d)	0.96 (0.90–1.03)	0.96 (0.87–1.05)	0.96 (0.88–1.06)	0.93
Yogurt (44.5 g/d)	0.98 (0.93–1.04)	0.96 (0.87–1.07)	0.99 (0.92–1.07)	0.64
Fruit and vegetables (172 g/d)	0.99 (0.92–1.05)	1.06 (0.97–1.16)	0.93 (0.85–1.03)	0.06
Energy intake (2.10 MJ/d)	1.01 (0.95–1.08)	1.06 (0.96–1.16)	0.98 (0.89–1.07)	0.22
Protein (2.7% energy)	1.01 (0.95–1.09)	1.03 (0.92–1.16)	1.01 (0.92–1.10)	0.74
Protein from dairy products (1.3% energy)	1.00 (0.94–1.06)	1.03 (0.94–1.13)	0.97 (0.89–1.06)	0.38
Carbohydrate (6.8% energy)	0.98 (0.92–1.05)	1.03 (0.93–1.13)	0.95 (0.87–1.04)	0.26
Total fat (5.4% energy)	1.01 (0.95–1.07)	0.99 (0.90–1.09)	1.02 (0.94–1.11)	0.61
Saturated fat (3.0% energy)	1.00 (0.94–1.06)	0.98 (0.89–1.08)	1.01 (0.92–1.09)	0.73
Monounsaturated fat (2.1% energy)	1.01 (0.95–1.08)	1.01 (0.92–1.11)	1.02 (0.94–1.11)	0.88
Polyunsaturated fat (1.7% energy)	1.01 (0.95–1.07)	0.98 (0.90–1.08)	1.03 (0.95–1.13)	0.42
n-6 fatty acids (1.8% energy)^d^	0.97 (0.90–1.05)	0.98 (0.89–1.08)	0.95 (0.83–1.10)	0.77
n-3 fatty acids (0.27% energy)^d^	1.01 (0.94–1.09)	1.02 (0.92–1.13)	1.01 (0.91–1.13)	0.88
Ratio n-6:n-3 (3.7)^d^	0.99 (0.92–1.06)	0.97 (0.89–1.06)	1.02 (0.90–1.16)	0.55
Alcohol (21.6 g/d)	1.00 (0.93–1.06)	0.97 (0.88–1.07)	1.02 (0.93–1.11)	0.49
Dietary fibre (6.0 g/d)	0.97 (0.90–1.04)	1.04 (0.94–1.14)	0.92 (0.83–1.02)	0.09
Retinol (1055 μg/d)	1.02 (0.96–1.09)	1.04 (0.96–1.12)	1.00 (0.90–1.12)	0.62
Carotene (1568 μg/d)^d^	1.01 (0.94–1.08)	1.05 (0.96–1.15)	0.94 (0.82–1.07)	0.17
Lycopene (1978 μg/d)^d^	0.96 (0.89–1.03)	0.94 (0.84–1.05)	0.97 (0.88–1.07)	0.68
Vitamin B6 (0.67 mg/d)	1.05 (0.98–1.13)	1.09 (0.98–1.21)	1.04 (0.94–1.15)	0.54
Folate (90 μg/d)	1.01 (0.94–1.09)	1.05 (0.95–1.16)	1.00 (0.90–1.10)	0.46
Vitamin B12 (3.97 μg/d)	1.03 (0.96–1.09)	1.06 (0.98–1.15)	0.99 (0.89–1.09)	0.28
Vitamin C (52.8 mg/d)	1.02 (0.95–1.08)	1.02 (0.92–1.12)	1.02 (0.94–1.11)	0.91
Vitamin D (2.77 μg/d)	1.01 (0.95–1.07)	0.92 (0.83–1.03)	1.06 (0.98–1.15)	0.04
Vitamin E (4.9 mg/d)	1.02 (0.95–1.10)	1.01 (0.91–1.11)	1.05 (0.94–1.18)	0.55
Calcium (283 mg/d)	1.03 (0.95–1.11)	1.05 (0.94–1.18)	0.99 (0.88–1.11)	0.46
Iron (3.9 mg/d)	1.00 (0.93–1.08)	1.00 (0.89–1.12)	1.02 (0.92–1.15)	0.75
Magnesium (91 mg/d)	0.98 (0.90–1.06)	1.02 (0.91–1.14)	0.96 (0.85–1.08)	0.48
Selenium (34.5 μg/d)	1.00 (0.94–1.07)	0.95 (0.85–1.07)	1.04 (0.96–1.13)	0.22
Zinc (2.53 mg/d)	0.99 (0.91–1.07)	1.03 (0.92–1.15)	0.96 (0.85–1.08)	0.39

a Conditional logistic regression adjusted for age, BMI, socioeconomic, smoking and marital status, diabetes and energy intake.

b Number of cases and controls.

c Test of heterogeneity of trends between cohort studies (mostly clinically-detected disease) and ProtecT (PSA-detected disease).

d Unknown for some participants.

**Table 6:** Odds ratios for prostate cancer with dietary intake by disease stage

*Food or nutrient intake (1 s.d.)*^a^	*Localised or advanced stage,* N=*1503/2418*^b^^c^	*Localised stage,* N=*1277/1952*^c^	*Advanced stage,* N=*226/466*^c^	P *value for heterogeneity by disease stage*^d^
Red meat (31.3 g/d)	1.01 (0.94–1.09)	1.04 (0.96–1.13)	0.83 (0.66–1.04)	0.06
Processed meat (21.8 g/d)	1.00 (0.93–1.08)	1.01 (0.93–1.09)	0.99 (0.80–1.24)	0.92
Red and processed meat (39.8 g/d)	1.01 (0.93–1.09)	1.04 (0.96–1.13)	0.85 (0.67–1.07)	0.11
Poultry (25.1 g/d)	0.99 (0.92–1.06)	0.99 (0.91–1.07)	0.99 (0.81–1.21)	0.96
White fish (17.4 g/d)	1.00 (0.93–1.08)	0.99 (0.92–1.08)	1.10 (0.90–1.35)	0.37
Oily fish (21.7 g/d)	1.02 (0.95–1.10)	1.02 (0.94–1.10)	1.06 (0.87–1.29)	0.71
Milk (144 g/d)	1.02 (0.95–1.11)	1.01 (0.93–1.10)	1.11 (0.92–1.35)	0.36
Cheese (17.2 g/d)	0.93 (0.86–1.01)	0.90 (0.83–0.99)	1.03 (0.84–1.26)	0.25
Yogurt (44.5 g/d)	0.96 (0.89–1.03)	0.96 (0.89–1.03)	1.01 (0.80–1.27)	0.65
Fruit and vegetables (172 g/d)	0.98 (0.91–1.06)	0.97 (0.89–1.05)	1.11 (0.90–1.36)	0.23
Energy intake (2.10 MJ/d)	1.03 (0.95–1.11)	1.00 (0.92–1.08)	1.23 (1.00–1.51)	0.07
Protein (2.7% energy)	1.01 (0.93–1.09)	1.01 (0.92–1.11)	1.00 (0.79–1.27)	0.96
Protein from dairy products (1.3% energy)	0.97 (0.90–1.04)	0.94 (0.87–1.02)	1.09 (0.88–1.34)	0.21
Carbohydrate (6.8% energy)	0.98 (0.91–1.06)	0.98 (0.90–1.07)	1.04 (0.83–1.29)	0.64
Total fat (5.4% energy)	1.05 (0.97–1.13)	1.04 (0.96–1.13)	1.03 (0.84–1.27)	0.93
Saturated fat (3.0% energy)	1.04 (0.96–1.12)	1.02 (0.94–1.11)	1.08 (0.88–1.34)	0.62
Monounsaturated fat (2.1% energy)	1.04 (0.97–1.12)	1.05 (0.97–1.13)	0.99 (0.81–1.22)	0.64
Polyunsaturated fat (1.7% energy)	1.04 (0.96–1.11)	1.05 (0.97–1.14)	0.94 (0.77–1.16)	0.33
n-6 fatty acids (1.8% energy)^b^	0.98 (0.89–1.07)	0.99 (0.90–1.10)	0.91 (0.73–1.13)	0.48
n-3 fatty acids (0.27% energy)^b^	1.01 (0.93–1.10)	1.02 (0.93–1.12)	0.96 (0.75–1.22)	0.67
Ratio n-6:n-3 (3.7)^b^	1.01 (0.92–1.10)	1.01 (0.92–1.12)	0.97 (0.79–1.20)	0.70
Alcohol (21.6 g/d)	0.97 (0.90–1.05)	0.97 (0.90–1.06)	0.93 (0.74–1.17)	0.72
Dietary fibre (6.0 g/d)	0.98 (0.90–1.06)	0.96 (0.88–1.05)	1.14 (0.91–1.43)	0.17
Retinol (1055 μg/d)	1.03 (0.95–1.11)	1.02 (0.93–1.11)	1.09 (0.91–1.29)	0.50
Carotene (1568 μg/d)^b^	1.02 (0.93–1.11)	1.00 (0.91–1.11)	1.06 (0.84–1.35)	0.66
Lycopene (1978 μg/d)^b^	0.98 (0.90–1.06)	0.98 (0.90–1.07)	0.93 (0.72–1.19)	0.67
Vitamin B6 (0.67 mg/d)	1.02 (0.94–1.11)	1.02 (0.93–1.12)	1.08 (0.84–1.38)	0.70
Folate (90 μg/d)	1.01 (0.93–1.10)	1.00 (0.92–1.10)	1.08 (0.85–1.38)	0.58
Vitamin B12 (3.97 μg/d)	1.04 (0.97–1.12)	1.04 (0.96–1.13)	1.05 (0.86–1.29)	0.93
Vitamin C (52.8 mg/d)	1.00 (0.92–1.08)	1.00 (0.92–1.09)	0.99 (0.79–1.24)	0.93
Vitamin D (2.77 μg/d)	1.02 (0.95–1.09)	1.03 (0.96–1.12)	0.94 (0.74–1.19)	0.44
Vitamin E (4.9 mg/d)	1.03 (0.94–1.13)	1.04 (0.94–1.15)	0.97 (0.78–1.21)	0.57
Calcium (283 mg/d)	0.97 (0.88–1.07)	0.96 (0.86–1.06)	1.06 (0.82–1.37)	0.47
Iron (3.9 mg/d)	0.99 (0.91–1.09)	1.02 (0.92–1.12)	0.87 (0.67–1.13)	0.28
Magnesium (91 mg/d)	0.97 (0.89–1.07)	0.96 (0.87–1.06)	1.08 (0.83–1.41)	0.42
Selenium (34.5 μg/d)	1.00 (0.93–1.08)	0.99 (0.90–1.08)	0.99 (0.83–1.17)	0.99
Zinc (2.53 mg/d)	1.00 (0.91–1.10)	1.01 (0.91–1.12)	0.96 (0.73–1.26)	0.75

a Conditional logistic regression adjusted for age, BMI, smoking, marital status, diabetes, socioeconomic status and energy intake.

b Stage unknown for 214 cases.

c Number of cases and controls.

d Test of heterogeneity of trends between localised and advanced disease.
