# Erratum: Prostate cancer risk related to foods, food groups, macronutrients and micronutrients derived from the UK Dietary Cohort Consortium food diaries

**DOI:** 10.1038/ejcn.2017.17

**Published:** 2017-03-15

**Authors:** J A Lane, S E Oliver, P N Appleby, M A H Lentjes, P Emmett, D Kuh, A Stephen, E J Brunner, M J Shipley, F C Hamdy, D E Neal, J L Donovan, K-T Khaw, T J Key

**Keywords:** Epidemiology, Prostate cancer, Prognosis, Nutrition

**Correction to:** European Journal of Clinical Nutrition (2017) 71, 274–283; doi:10.1038/ejcn.2016.162; published online 28 September 2016

**Updated online 15 February 2017:** This article was originally published under a CC BY-NC-SA 4.0 license, but has now been made available under a CC BY 4.0 license. The PDF and HTML versions of the paper have been modified accordingly.

